# Selective Phospholipase A2 Gene Expression in Peri-Implantitis: An Intra-Individual Paired Gingival Tissue Study

**DOI:** 10.3390/ijms27198717

**Published:** 2026-09-29

**Authors:** Alicja Baranowska, Marzena Dominiak, Jacek Matys, Jakub Hadzik, Tomasz Gedrange, Julita Kulbacka, Dagmara Baczyńska, Barbara Sterczała, Artur Pitułaj, Mieszko Więckiewicz, Andrzej Małysa, Paweł Kubasiewicz-Ross

**Affiliations:** 1 Oral Surgery Department, Wroclaw Medical University, 50-425 Wroclaw, Poland; alicja.baranowska@umw.edu.pl (A.B.); marzena.dominiak@umw.edu.pl (M.D.); jakub.hadzik@umw.edu.pl (J.H.); barbara.sterczala@umw.edu.pl (B.S.); artur.pitulaj@umw.edu.pl (A.P.); 2 Department of Molecular and Cellular Biology, Wroclaw Medical University, 50-556 Wroclaw, Poland; julita.kulbacka@umw.edu.pl (J.K.); dagmara.baczynska@umw.edu.pl (D.B.); 3 Department of Experimental Dentistry, Wroclaw Medical University, 50-425 Wroclaw, Poland; mieszko.wieckiewicz@umw.edu.pl (M.W.); andrzej.malysa@umw.edu.pl (A.M.)

**Keywords:** peri-implantitis, phospholipase A2, gene expression

## Abstract

Phospholipase A2 (PLA2) enzymes participate in lipid metabolism and inflammatory signaling, but the expression of individual PLA2-family genes in peri-implantitis remains poorly characterized. This exploratory study compared the expression of *PLA2G7*, *PLA2G4A*, *PLA2G6*, and *PLA2G12A* in paired gingival samples from 19 patients with advanced peri-implantitis requiring implant removal. Inflamed tissue and clinically non-inflamed local reference tissue were collected adjacent to the same affected implant. Gene expression was assessed by quantitative reverse transcription polymerase chain reaction and normalized to on glyceraldehyde-3-phosphate dehydrogenase (*GAPDH*). Paired expression differences were evaluated using Wilcoxon signed-rank tests with Benjamini–Hochberg adjustment. Complete paired data were available for 19 patients for three genes and 18 patients for *PLA2G4A*. *PLA2G7* expression was higher in inflamed tissue in 15 of 19 patients, with a median fold change of 5.86 (interquartile range, 1.29–17.36; adjusted *p* = 0.0247). No significant paired differences were detected for the other three genes. In comparisons across genes, the fold change for *PLA2G7* exceeded that for *PLA2G12A* after Holm adjustment, but did not differ significantly from those for *PLA2G4A* or *PLA2G6*. These findings suggest an association between advanced peri-implantitis and increased tissue-level *PLA2G7* expression. Further studies are needed to establish its functional significance and confirm these preliminary findings.

## 1. Introduction

Dental implants have become a standard and predictable method of dental treatment. Their use has grown substantially over the last two decades. In adults aged 50 years or older, the prevalence of having at least one dental implant increased from 1.3% in 1999–2004 to 8.4% in 2015–2020 [1]. As the number of implant-treated patients increases, biological complications become more clinically relevant. One of the most common is peri-implantitis. Its prevalence has been estimated at 19.5% at the patient level and 12.5% at the implant level [2].

In the initiation of peri-implantitis, alongside bacterial factors, dysregulation of the innate immune response plays a major role. Recent studies have demonstrated substantial changes in genes involved in inflammation, immune regulation, and tissue remodeling in peri-implantitis [3]. Moreover, on the cell level, further increase in immune-cell infiltration, heterogeneous fibroblast populations, enhanced neutrophil recruitment, and changes in macrophage-lineage cells differentiation has been reported [4]. These findings support the concept that altered cellular physiology is an important component of peri-implantitis etiopathogenesis.

Host-related inflammatory changes may also involve mediators linking clinical situation and immune response. In periodontitis, Wiyanto et al. reported higher gingival crevicular fluid interleukin-18 (IL-18) levels in patients with more severe disease, supporting the relationship between local cytokine activity and periodontal tissue destruction. Suragimath et al. further demonstrated significant changes in serum and salivary leptin following non-surgical periodontal therapy, suggesting that periodontal inflammation may also influence mediators involved in immune-metabolic regulation [5,6]. At the tissue level, altered gingival expression of genes involved in oxidative and inflammatory responses has also been demonstrated in periodontitis [7]. Systemic inflammatory and immune-metabolic biomarkers have also been associated with disease severity in advanced periodontitis [8].

Lipid metabolism may represent one of the less explored components of this response. Membrane phospholipids provide substrates for the formation of several biologically active lipid mediators. Phospholipase A2 (PLA2) enzymes hydrolyze membrane phospholipids and modify the availability of fatty acids and lysophospholipids. In periodontitis, lipidomic alterations and increased expression of secretory phospholipase A2-IIA have already been demonstrated [9]. However, corresponding data for peri-implantitis remain scarce.

The PLA2 superfamily is functionally heterogeneous. Individual enzymes differ in catalytic mechanisms, cellular localization, substrate preference, and biological function [10]. Therefore, increased inflammatory activity should not be expected to produce a uniform change in all PLA2-family genes. Analysis of individual PLA2 members may provide more specific information than assessment of phospholipase activity as a single pathway.

To date, the relative expression of selected PLA2 genes in human peri-implantitis tissue has not been well characterized. In addition, most available molecular studies compare samples obtained from separate groups of individuals [3,4]. An intra-individual paired design may reduce part of this biological variability and provide a more direct assessment of local disease-associated changes.

Therefore, the aim of the present study was to compare the expression of *PLA2G7*, *PLA2G4A*, *PLA2G6*, and *PLA2G12A* in gingival tissue obtained from peri-implantitis sites and from a clinically non-inflamed gingival area in the immediate vicinity of the same implant in the same patients.

## 2. Results

### 2.1. Patient Characteristics

The study included 19 patients, comprising 11 women (57.9%) and 8 men (42.1%). The mean age was 57.6 ± 16.0 years. The median age was 57 years (interquartile range [IQR], 42.5–72.5), with an overall range of 32–79 years. The median patient-level mean probing depth (PD) was 6.50 mm. Peri-implant soft-tissue measurements varied considerably, with height of keratinized tissue (HKT) recorded as zero in eight patients (42.1%). The median approximal plaque index (API) was 20.0%. Screw-retained restorations predominated. All implants were two-piece titanium implants with sandblasted, large-grit, acid-etched (SLA) surfaces. Detailed clinical and prosthetic characteristics, including the duration of implant function, are presented in Table 1.

Regarding general health of the cohort type 2 diabetes was recorded in four patients (21.1%; No. 1, 5, 10, and 14), and hypertension in four patients (21.1%; No. 4, 5, 14, and 19). Patients No. 5 and No. 14 had both conditions. Asthma was recorded in patient No. 7 and allergy in patient No. 9. Smoking was reported by patients No. 1 and No. 5.

### 2.2. Study Sample and Data Completeness

Complete paired expression data were available for *PLA2G7*, *PLA2G6*, and *PLA2G12A* in all 19 patients. For *PLA2G4A*, one measurement was unavailable because of a technical assay failure; therefore, the *PLA2G4A* analysis included 18 paired samples. Individual relative quantification (RQ) values are presented in Table 2.

### 2.3. Expression of Individual PLA2 Genes

*PLA2G7* expression was higher in peri-implantitis tissue in 15 of 19 patients. The median RQ was 5.86 (IQR, 1.29–17.36) and the geometric mean RQ was 4.96 (95% Confidence Interval (CI), 2.00–11.96). The paired difference was statistically significant (*p* = 0.0062; *q* = 0.0247). Individual RQ values ranged from 0.09 to 165.18 (Table 2).

For *PLA2G4A*, the median RQ was 0.99 (IQR, 0.73–1.08), with no statistically significant paired difference (*p* = 0.6705; *q* = 0.7983). For *PLA2G6*, the median RQ was 1.08 (IQR, 0.42–2.23) and the paired difference was also not statistically significant (*p* = 0.7983; *q* = 0.7983) (Table 3).

*PLA2G12A* expression was lower in peri-implantitis tissue in 15 of 19 patients. The median RQ was 0.76 (IQR, 0.40–1.00), with no statistically significant paired difference (*p* = 0.0799; *q* = 0.1598). Geometric means and their 95% confidence intervals for all four genes are presented in Table 3.

### 2.4. Comparison of Expression Across PLA2 Genes

**Figure 1:**
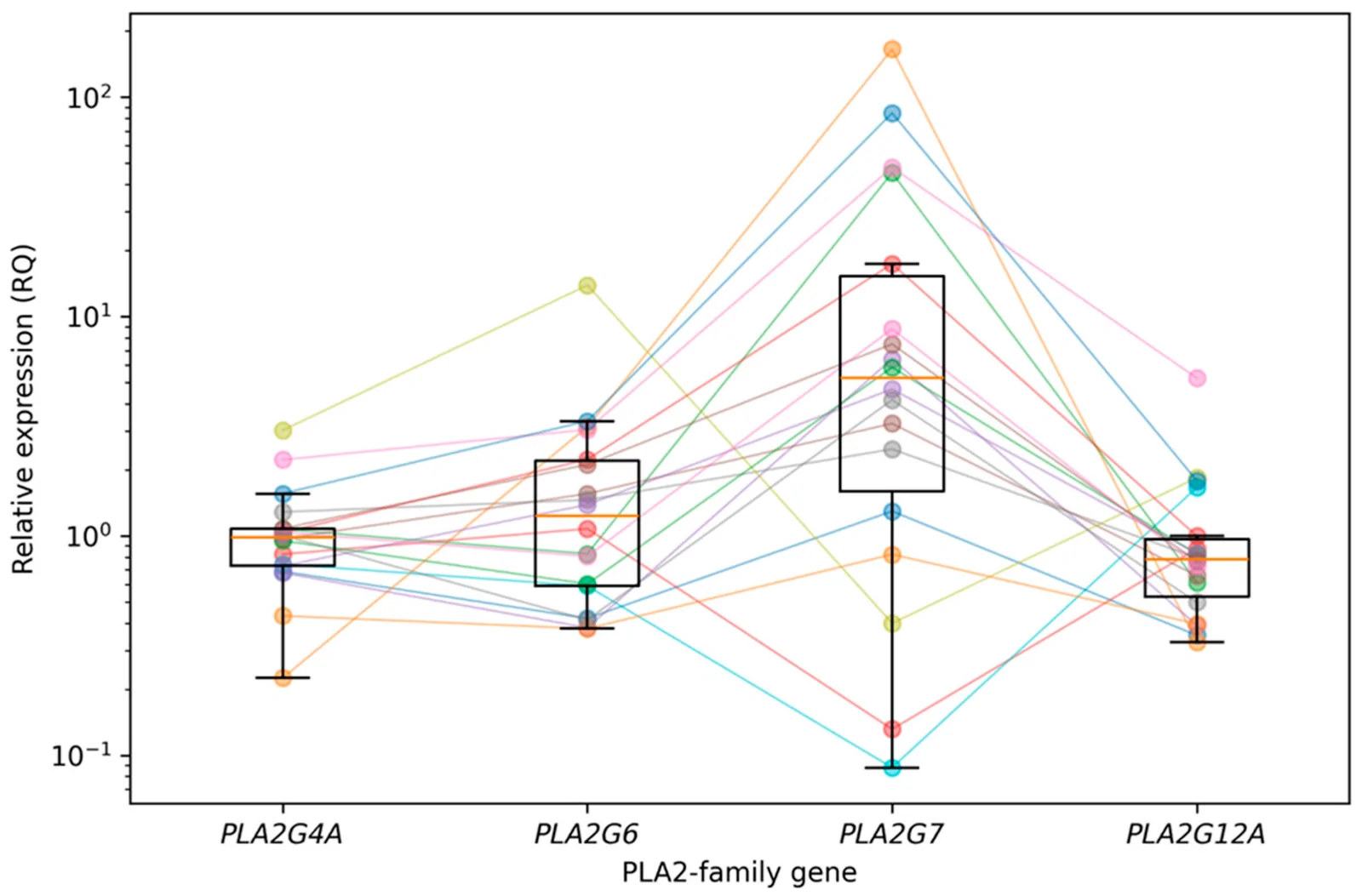
Individual relative expression profiles and box plots of the four PLA2-family. RQ, relative quantification. Colored markers indicate patient-level values, and lines connect measurements from the same participant. Colors distinguish participant profiles and do not represent clinical groups. Boxes show the interquartile ranges, and orange horizontal lines indicate the medians. The vertical axis uses a logarithmic scale.

Relative expression differed significantly across the four investigated PLA2-family genes. In the complete-case analysis of 18 patients, the Friedman test showed a significant overall difference (χ^2^(3) = 16.27, *p* < 0.001, Kendall’s W = 0.301) (Figure 1). Post hoc comparisons showed a greater fold change for PLA2G7 than for PLA2G12A (Holm-adjusted *p* = 0.0461). Differences between PLA2G7 and PLA2G4A or PLA2G6 were not statistically significant after adjustment (Holm-adjusted *p* = 0.0602 and 0.0729, respectively). No other pairwise comparisons were statistically significant after adjustment.

## 3. Discussion

The biological variability of peri-implantitis appears to extend beyond differences in the intensity of inflammation. Recent tissue-based studies indicate changes in both the composition and functional state of cells within peri-implant lesions when compared to physiological condition. Malmqvist et al. demonstrated enrichment of B cells and an enhanced inflammatory mediator profile directly in peri-implantitis tissues [11]. Mo et al. identified a predominance of inflammation, promoting fibroblast-neutrophil interactions [12]. Yamaguchi et al. further demonstrated collagen-degrading activity in fibroblasts isolated directly from peri-implantitis lesions [13], while Park et al. reported enhanced neutrophilic signaling related to C-X-C motif chemokine ligands 5 and 8 (CXCL5 and CXCL8)- in peri-implantitis tissue [14]. These observations suggest that altered cellular activity, rather than inflammation alone, may contribute to the destructive phenotype of the disease.

*PLA2G7* expression showed the clearest change. Expression was higher at the peri-implantitis site in 15 of 19 patients and remained significant after correction for multiple comparisons. In contrast, the expression of *PLA2G4A* and *PLA2G6* showed no consistent directional change, while, *PLA2G12A* was lower in 15 of 19 patients.

Despite increasing interest in molecular mechanisms of peri-implantitis, the available literature remains small. Spinell et al. identified only nine eligible in vivo omics studies, of which five involved human material [15]. Martin et al. identified changes in pathways related to intracellular signaling, phagocytosis, and oxidative stress [16]. More recently, Dionigi et al. demonstrated transcriptional alterations involving collagen organization and angiogenesis [17]. None of these studies investigated PLA2 genes transcription changes. Therefore, the present analysis addresses a relatively narrow but largely unexplored molecular pathway.

The intra-individual design is another relevant aspect of the present study. Gene expression may be influenced by age, systemic conditions and other host-related factors. Comparison of diseased and non-inflamed tissue from the same patient reduces part of this variability. Paired sampling reduces variation related to age and other patient characteristics, although systemic disease may affect inflamed and reference tissues differently. PLA2 enzymes are involved in lipid metabolism and inflammatory signaling [10], making metabolic comorbidities relevant to the interpretation of their expression. Type 2 diabetes was recorded in four participants. *PLA2G7* expression was lower in inflamed tissue in patients Nos. 5, 10, 11, and 13. Patient No. 5 had diabetes and hypertension and reported smoking. Patient No. 1 also had diabetes and reported smoking, but showed higher *PLA2G7* expression in inflamed tissue. The cohort was too small to determine whether these conditions contributed to the observed expression patterns.

Peri-implant soft tissue phenotype represents an important component of the local peri-implant environment. Recent randomized clinical studies have demonstrated that soft tissue augmentation can produce a clinically relevant increase in peri-implant tissue thickness and that the magnitude of this biological response is influenced by baseline soft tissue characteristics [18,19]. These observations support considering local soft-tissue characteristics when interpreting tissue-level gene-expression findings. The discussion of other reports findings is particularly difficult in case of PLA2 transcription. However, several studies in periodontitis provide indirect biological support of present report. Shinohara et al. identified group II PLA2 activity directly in diseased human gingival tissue [20]. Lösche et al. subsequently showed that circulating lipoprotein-associated PLA2 activity was related to clinical periodontal inflammation and tissue destruction and decreased after periodontal treatment [21]. More recently, Isola et al. evaluated PLA2 in a randomized periodontal treatment trial and again linked this enzyme with the inflammatory burden of periodontitis [22]. These studies investigated different biological subjects and cannot be directly compared with local *PLA2G7* mRNA expression. They however might support the broader relevance of PLA2-related pathways in periodontitis. The recent findings of Jin et al. provide a particularly interesting mechanistic context for *PLA2G7* [23]. In their study, *Pla2g7* was found along the osteoclast developmental trajectory and consequently associated with bone resorption. Inhibition or deficiency of *Pla2g7* impaired osteoclast differentiation. This is relevant particularly to peri-implantitis because progressive osteoclastic bone destruction is a defining feature of the disease.

No statistically significant paired expression differences were detected for *PLA2G4A* or *PLA2G6*, although the small sample limits interpretation of these findings. *PLA2G12A* expression was lower in most patients, although the difference was not statistically significant. Recent report has shown that PLA2G12A can promote pathogenic pathogenic T helper 17 (Th17) cell responsesresponses through extracellular-vesicle lipid signaling [24]. Our findings do not reproduce this direction of change in peri-implantitis tissue. The discrepancy may reflect tissue-specific regulation, differences in cellular composition, or the advanced stage of the lesions included in our cohort.

Several limitations of the present study should be considered. The cohort included only 19 patients, and one *PLA2G4A* expression measurement was unavailable. These findings should be considered preliminary because of the small sample and exploratory design. Non-significant results do not establish the absence of expression changes. All patients had advanced peri-implantitis requiring implant removal, and the findings cannot automatically be extrapolated to early or moderate disease. Higher *PLA2G7* expression in whole-tissue samples could reflect a greater proportion of inflammatory cells, including macrophages, increased expression within individual cells or both. The present analysis could not distinguish between these explanations. The reference sample was obtained from clinically non-inflamed gingiva in the immediate vicinity of the affected implant and therefore should not be considered equivalent to tissue surrounding a healthy implant. Subclinical molecular alterations within the local peri-implant environment cannot be excluded. Such alterations may have attenuated the observed expression differences between inflamed and local reference tissues. Normalization relied on glyceraldehyde-3-phosphate dehydrogenase (*GAPDH*) as the sole reference gene, and its expression stability between inflamed and local reference tissues was not established across the cohort. This may have affected the estimated PLA2 fold changes. Individual *GAPDH* quantification cycle (Cq) values for all 19 participants are provided in Supplementary Figure S1. Finally, mRNA expression does not directly demonstrate increased protein concentration or enzymatic activity. No corresponding PLA2 protein measurement or lipidomic analysis was performed. The cross-sectional tissue comparison also prevents conclusions about causality. It remains unclear whether increased *PLA2G7* expression contributes to peri-implant tissue destruction or develops as a consequence of an established inflammatory lesion. Within these limitations, the present findings support an association between advanced peri-implantitis and higher tissue-level *PLA2G7* mRNA expression. However, its fold change did not differ significantly from those of *PLA2G4A* or *PLA2G6* after Holm adjustment. Together with recent evidence linking *Pla2g7* to osteoclast differentiation and bone resorption, this result identifies *PLA2G7* as a potentially relevant molecular component of peri-implantitis and supports further tissue-level investigation.

## 4. Materials and Methods

### 4.1. Study Design

The present report describes an exploratory, prospective molecular substudy nested within the nonrandomized, single-group PERI-EDU clinical study. The molecular analysis used an intra-individual paired design. Patients were recruited and treated at the Department of Oral Surgery, Wroclaw Medical University, Wroclaw, Poland.

The present molecular analysis included 19 patients with active advanced peri-implantitis requiring implant explantation. No a priori sample-size calculation was performed for this exploratory molecular substudy. In each participant, inflamed peri-implant tissue was compared with clinically non-inflamed gingival tissue obtained from the immediate vicinity of the same affected implant.

The first 13 participants in the present cohort were included in the preliminary PERI-EDU report, which focused on clinical and radiographic findings, systemic inflammatory markers and scanning electron microscopy and energy-dispersive X-ray spectroscopy (SEM/EDX)-defined corrosion-related changes [25]. The present molecular substudy includes six additional participants, bringing the cohort to 19. No tissue-level molecular or PLA2 gene-expression data were reported in the previous publication. The study is reported according to the Strengthening the Reporting of Observational Studies in Epidemiology (STROBE) statement, with consideration of the Strengthening the Reporting of Observational Studies in Epidemiology–Molecular Epidemiology STROBE-ME recommendations for molecular epidemiology studies [26,27]. Participant screening, eligibility assessment, paired tissue sampling, and inclusion in the molecular analyses are summarized in Supplementary Figure S1.

The study was conducted in accordance with the Declaration of Helsinki. The protocol was approved by the Bioethics Committee at Wroclaw Medical University, Poland (KB 601/2024). The study was registered at ClinicalTrials.gov under identifier NCT07012915. All participants provided written informed consent for the surgical procedure and participation in the study. The study was conducted in accordance with the General Data Protection Regulation (GDPR).

### 4.2. Eligibility Criteria

Adult patients of both sexes were screened for participation. Patients were eligible if they had undergone previous dental implant treatment and presented with an implant affected by active advanced peri-implantitis requiring explantation.

Active peri-implantitis was defined as probing depth (PD) ≥ 6 mm at the affected implant site together with positive bleeding on probing. Advanced peri-implantitis was defined as peri-implant bone loss radiographically exceeding 66% of the implant length. Implant removal was considered clinically indicated when both criteria were fulfilled.

If more than one implant was present, the remaining implants were required to be clinically healthy, defined as PD ≤ 3 mm and negative bleeding on probing.

Exclusion criteria were age < 18 years, pregnancy, general contraindications to oral surgical treatment, local contraindications to implant explantation, severe systemic disease associated with increased surgical risk, autoimmune disease, and current or previous treatment with bisphosphonates or other antiresorptive medications.

### 4.3. Clinical and Radiographic Examination

All participants underwent clinical examination before explantation. PD was measured at four peri-implant surfaces: buccal, lingual or palatal, mesial, and distal. Bleeding on probing was recorded. Implant mobility was assessed clinically when present. Keratinized tissue height (HKT) was measured using a periodontal probe scalable in 1 mm.

Cone-beam computed tomography (CBCT) was performed as part of the diagnostic assessment. Imaging was performed using the Planmeca Viso G7 system (Planmeca, Helsinki, Finland), and scans were analyzed using Romexis software version 6.5.2 (Planmeca, Helsinki, Finland).

Peri-implant bone loss was assessed at the buccal, lingual or palatal, mesial, and distal surfaces. Bone loss was expressed as a percentage of implant length and calculated as the ratio between the vertical distance from the implant platform to the bottom of the bone defect and the total implant length.

### 4.4. Implant Explantation and Soft-Tissue Biopsy Collection

All implant explantations were performed as standard oral surgical procedures by two investigators selected from A.B., P.K.-R., B.S., and A.P. Investigator pairs were formed at random, and patients were randomly assigned to these pairs. Both biopsy sites were selected jointly by the two investigators, with the final decision reached by consensus. The prosthetic restoration was removed before surgery. Following oral disinfection, local anesthesia was administered using 4% articaine with epinephrine 1:200,000. A full-thickness mucoperiosteal flap was elevated to expose the peri-implant defect. During implant explantation, soft-tissue material was collected from the peri-implant surgical field adjacent to the affected implant. The tissue-sampling strategy was guided by ethical considerations and the scope of the Bioethics Committee approval. Accordingly, both specimens were obtained within the existing surgical field, without establishing an additional surgical site elsewhere in the oral cavity. The inflamed specimen was collected as part of the clinically indicated removal of the most severely inflamed soft tissues during implant explantation and preparation for wound closure. It comprised a soft-tissue fragment comprising epithelium and underlying connective tissue of the buccal flap overlying the peri-implant bone defect. The paired local reference specimen was obtained from clinically non-inflamed tissue on the palatal or lingual aspect of the same implant, from an area overlying preserved bone. Reference-site selection was guided by the corresponding palatal or lingual PD and the size and configuration of the bone defect judged on the CBCT basis. The distance between the gingival margin and the reference sampling site exceeded the PD measured on that aspect. The reference site was judged clinically non-inflamed on the basis of the absence of visible erythema and swelling. This assessment was made jointly by the two investigators and combined with the anatomical criteria described above. Both specimens included epithelium and underlying connective tissue and were processed separately for tissue-level gene-expression analysis. A similar tissue-sampling approach was described by Olander et al. [28], who demonstrated that small peri-implant mucosal biopsies obtained in close proximity to implant abutments can be successfully used for quantitative polymerase chain reaction (qPCR)-based gene-expression analysis. Samples intended for molecular analysis were transferred to the Department of Molecular and Cellular Biology, Faculty of Pharmacy, Wroclaw Medical University, and stored at −80 °C until RNA isolation. The affected implant was removed using reverse torque. The peri-implant defect was subsequently curetted and irrigated with sterile saline, and the wound was closed with single monofilament sutures.

### 4.5. RNA Isolation

Gene-expression analysis was performed directly on material obtained from the collected gingival biopsies. For total RNA isolation, tissue fragments weighing 5–15 mg were used.

The first stage of homogenization was performed in Lysis Buffer RL (EURx Ltd., Gdańsk, Poland) supplemented with β-mercaptoethanol using MagNA Lyser Green Beads B (Roche Diagnostics GmbH, Mannheim, Germany) at 1 °C and 1500 rpm for 10 min. The samples were then homogenized and pre-purified using homogenization mini columns (EURx Ltd., Gdańsk, Poland), followed by digestion with proteinase K (EURx Ltd., Gdańsk, Poland). Final RNA purification was performed on spin columns using the GeneMATRIX Universal RNA Purification Kit (EURx Ltd., Gdańsk, Poland) and DNase I (EURx Ltd., Gdańsk, Poland) according to the manufacturer’s recommendations.

RNA concentration and purity were determined spectrophotometrically. Across the 38 tissue samples, RNA concentrations ranged from 24.88 to 434.60 ng/µL, and A260/A280 ratios ranged from 1.910 to 2.162. RNA integrity was not assessed. Individual RNA concentration and purity measurements are provided in Supplementary Figure S1. RNA samples were then stored at −80 °C or used directly for reverse transcription reactions.

### 4.6. Quantitative RT-PCR

Reverse transcription reactions were performed using up to 2 µg of extracted RNA per 20 µL reaction and the High Capacity cDNA Reverse Transcription Kit (Applied Biosystems, Foster City, CA, USA) according to the manufacturer’s recommendations. For samples with insufficient RNA concentration to provide 2 µg, the maximum permissible RNA volume of 14.2 µL was used. Two parallel 20 µL reverse-transcription reactions were performed for each sample. The products were pooled and diluted threefold with water before quantitative PCR using Fast Probe qPCR Master Mix (EURx Ltd., Gdańsk, Poland) and TaqMan Gene Expression Assays (Thermo Fisher Scientific, Waltham, MA, USA). All reactions were performed in technical triplicates using an Optical Real-Time PCR Thermocycler (Biometra GmbH, Göttingen, Germany). The cycling conditions were 3 min at 95 °C, followed by 40 cycles of 10 s at 95 °C and 30 s at 60 °C. *GAPDH* was used as the sole reference gene, consistent with its previous use for normalization in quantitative reverse transcription polymerase chain reaction (RT-qPCR) analyses of human gingival tissue in periodontitis [7]. Its expression stability between inflamed and local reference tissues was not established across the present cohort.

Target-gene threshold cycle (Ct) values were normalized to the mean Ct of the *GAPDH* technical replicates from the same sample. The mean ΔCt of the paired local reference tissue was used as the calibrator. RQ was calculated separately for each technical replicate of the inflamed tissue, and these values were averaged arithmetically to obtain one RQ value per patient and gene. Relative expression values were summarized as medians with interquartile ranges and geometric means with 95% confidence intervals.

The following TaqMan Gene Expression Assays (Thermo Fisher Scientific, Waltham, MA, USA) were used: *GAPDH* (Hs99999905_m1), *PLA2G4A* (Hs00996912_m1), *PLA2G6* (Hs00895669_m1), *PLA2G7* (Hs00965837_m1), and *PLA2G12A* (Hs04976755_s1). For *GAPDH*, no-template controls and reverse-transcription blanks prepared without RNA were used. These yielded either no detectable amplification or late Cq values of 35.96–37.04. No-reverse-transcriptase (no-RT) control results were unavailable. Individual *GAPDH* and target-gene Cq values, ΔCt values, and the available negative-control results are provided in Supplementary Figure S1.

### 4.7. Statistical Analysis

RQ values were summarized as medians with interquartile ranges (Q1–Q3) and geometric means with 95% confidence intervals. Confidence intervals for geometric means were calculated using the percentile bootstrap method with 100,000 resamples at the patient level. These intervals were not adjusted for multiple comparisons. Within-patient differences were assessed using a two-sided Wilcoxon signed-rank test of log_2_ RQ against zero. Because four related PLA2 genes were analyzed, the Benjamini–Hochberg procedure was applied to control the false discovery rate. Both nominal p-values and adjusted q-values were reported. A two-sided *p*-value < 0.05 was considered nominally significant, while *q* < 0.05 was considered statistically significant after correction for multiple comparisons. Differences in relative expression across the four investigated PLA2-family genes were assessed using the Friedman test. This analysis was restricted to complete cases (*n* = 18). Kendall’s coefficient of concordance (W) was reported as an effect-size measure. Post hoc comparisons between genes were performed using two-sided paired Wilcoxon signed-rank tests on log_2_ RQ in the 18 patients with complete data. P-values were adjusted using the Holm procedure across all six pairwise comparisons. Statistical analyses were performed using STATISTICA software, version 13.3 (TIBCO Software Inc., Palo Alto, CA, USA). Bootstrap confidence intervals were calculated in Python (version 3.12.14; Python Software Foundation, Beaverton, OR, USA).

## 5. Conclusions

In patients with advanced peri-implantitis requiring explantation, *PLA2G7* was the only one of the four investigated genes to show significantly higher mRNA expression in inflamed tissue than in paired local reference tissue after correction for multiple comparisons. However, its fold change did not differ significantly from those of *PLA2G4A* or *PLA2G6* after Holm correction. These findings identify *PLA2G7* as a potentially relevant molecular component of peri-implantitis and support further studies assessing its protein expression, enzymatic activity, and relationship with local bone destruction.

## Figures and Tables

**Table 1:** Preoperative peri-implant and prosthetic characteristics of the study population.

Variable	Value, Median [Q1–Q3] or *n* (%)
Mean PD at the affected implant [mm]	6.50 [6.00–7.75]
HKT [mm]	1.0 [0.0–5.0]
API [%]	20.0 [10.0–32.5]
Screw-retained restoration, *n* (%)	12 (63.2)
Cement-retained restoration, *n* (%)	7 (36.8)
Duration of implant function [years]	11 [9,10,11,12,13]

Continuous variables are presented as median [Q1–Q3], and categorical variables as number (%). Mean PD was calculated separately for each patient from measurements at four peri-implant surfaces. PD, probing depth; API, approximal plaque index; HKT, height of keratinized tissue; Q1–Q3, first and third quartiles.

**Table 2:** Individual relative expression of the four PLA2 genes in peri-implantitis tissue relative to paired local reference gingival tissue.

Patient No.	*PLA2G7*	*PLA2G4A*	*PLA2G6*	*PLA2G12A*
1	1.294	0.688	0.420	0.352
2	16.449	NA	0.093	0.104
3	165.184	0.225	3.142	0.328
4	45.151	1.069	0.828	0.614
5	0.131	0.824	1.077	0.874
6	6.407	0.677	0.381	0.391
7	3.249	0.969	1.556	0.658
8	47.840	2.221	3.046	5.234
9	2.483	1.284	1.458	0.795
10	0.399	3.025	13.879	1.838
11	0.088	0.743	0.590	1.662
12	84.601	1.558	3.335	1.783
13	0.823	0.432	0.377	0.397
14	5.861	0.949	0.603	0.826
15	17.365	1.032	2.230	0.996
16	4.663	0.728	1.386	0.831
17	7.481	1.080	2.114	0.764
18	8.771	1.038	0.807	0.726
19	4.163	1.005	0.414	0.498

RQ, relative quantification. RQ = 2^−ΔΔCt^ relative to the paired clinically non-inflamed local reference sample. RQ > 1 indicates higher expression at the peri-implantitis site; RQ < 1 indicates lower expression relative to the local reference tissue. Individual RQ values are rounded to three decimal places for presentation. NA = not available.

**Table 3:** Summary of paired PLA2 gene-expression changes in peri-implantitis tissue relative to local reference tissue.

Gene	Median RQ [Q1–Q3]	Geometric Mean RQ [95% CI]	RQ > 1, *n*	RQ < 1, *n*	*p*/*q*
*PLA2G7*	5.86 [1.29–17.36]	4.96 [2.00–11.96]	15	4	0.0062/0.0247
*PLA2G4A*	0.99 [0.73–1.08]	0.94 [0.72–1.20]	9	9	0.6705/0.7983
*PLA2G6*	1.08 [0.42–2.23]	1.07 [0.66–1.76]	10	9	0.7983/0.7983
*PLA2G12A*	0.76 [0.40–1.00]	0.73 [0.51–1.06]	4	15	0.0799/0.1598

RQ, relative quantification; Q1–Q3, first and third quartiles; CI, confidence interval. RQ > 1 indicates higher expression in peri-implantitis tissue relative to the paired local reference tissue RQ < 1 indicates lower expression in peri-implantitis tissue relative to the paired local reference tissue. Confidence intervals refer to geometric means and were obtained by participant-level percentile bootstrap; they were not adjusted for multiple comparisons. Nominal *p*-values were obtained from two-sided Wilcoxon signed-rank tests on log_2_ RQ. The *q*-values represent Benjamini–Hochberg-adjusted *p*-values across the four genes.

## Data Availability

Individual Cq and ΔCt values, patient-level relative expression calculations, RNA concentration and purity measurements, and available negative-control results are provided in Supplementary Figure S1. Additional study data are available from the corresponding author on reasonable request, subject to ethical and privacy restrictions.

## Supplementary Materials

The following supporting information can be downloaded at https://www.mdpi.com/article/10.3390/ijms27198717/s1.

## Abbreviations

The following abbreviations are used in this manuscript:

API	approximal plaque index
CBCT	cone-beam computed tomography
cDNA	complementary DNA
EDX	energy-dispersive X-ray spectroscopy
GAPDH	glyceraldehyde-3-phosphate dehydrogenase
GDPR	General Data Protection Regulation
HKT	height of keratinized tissue
IQR	interquartile range
LIPSS	laser-induced periodic surface structures
Lp-PLA2	lipoprotein-associated phospholipase A2
PD	probing depth
PLA2	phospholipase A2
qPCR	quantitative polymerase chain reaction
RNA	ribonucleic acid
RQ	relative quantification
RT-PCR	reverse transcription polymerase chain reaction
SD	standard deviation
SEM	scanning electron microscopy
SLA	sandblasted, large-grit, acid-etched
STROBE	Strengthening the Reporting of Observational Studies in Epidemiology
